# Physeal growth arrest after tibial lengthening in achondroplasia: 23 children followed to skeletal maturity

**DOI:** 10.3109/17453674.2012.678802

**Published:** 2012-06-04

**Authors:** Sang-Heon Song, Mandar Vikas Agashe, Young-Jae Huh, Soon-Young Hwang, Hae-Ryong Song

**Affiliations:** ^1^Institute for Rare Diseases and Department of Orthopaedic Surgery; ^2^Department of Biostatistics, Korea University Medical Center, Guro Hospital, Seoul, Korea

## Abstract

**Background and purpose:**

Bilateral tibial lengthening has become one of the standard treatments for upper segment-lower segment disproportion and to improve quality of life in achondroplasia. We determined the effect of tibial lengthening on the tibial physis and compared tibial growth that occurred at the physis with that in non-operated patients with acondroplasia.

**Methods:**

We performed a retrospective analysis of serial radiographs until skeletal maturity in 23 achondroplasia patients who underwent bilateral tibial lengthening before skeletal maturity (lengthening group L) and 12 achondroplasia patients of similar height and age who did not undergo tibial lengthening (control group C). The mean amount of lengthening of tibia in group L was 9.2 cm (lengthening percentage: 60%) and the mean age at the time of lengthening was 8.2 years. The mean duration of follow-up was 9.8 years.

**Results:**

Skeletal maturity (fusion of physis) occurred at 15.2 years in group L and at 16.0 years in group C. The actual length of tibia (without distraction) at skeletal maturity was 238 mm in group L and 277 mm in group C (p = 0.03). The mean growth rates showed a decrease in group L relative to group C from about 2 years after surgery. Physeal closure was most pronounced on the anterolateral proximal tibial physis, with relative preservation of the distal physis.

**Interpretation:**

Our findings indicate that physeal growth rate can be disturbed after tibial lengthening in achondroplasia, and a close watch should be kept for such an occurrence—especially when lengthening of more than 50% is attempted.

Achondroplasia is the most common genetic form of dwarfism with the appearance of disproportionately short stature. Lower limb lengthening, especially bilateral tibial lengthening, has become one of the standard modalities of treatment for this body disproportion (Paley 1988, Cai et al. 2004). In recent years, there have been a number of reports about the benefits and complications of this long and arduous process (Paley 1990, Cai et al. 2004, Shyam et al. 2009, Venkatesh et al. 2009).

There is no consensus on the effects of limb lengthening on physeal growth. Some studies have shown no effect (Shapiro 1987, Lee et al. 2001), some have shown stimulation of physeal growth (Sabharwal et al. 2000), and some others have shown permanent cessation of physeal growth (Sharma et al. 1996). However, these studies have either been in animal models or in small non-comparative human series. Also, the indications for limb lengthening have either been congenital short femur and tibia or hemimelias. The effect of limb lengthening on future physeal growth has not been investigated in achondroplasia, where a massive amount of lengthening is required. This led us to retrospectively analyze all our patients who underwent tibial lengthening to look for signs of physeal damage.

## Patients and methods

We performed a retrospective study of all patients with achondroplasia who underwent bilateral tibial lengthening at our institutes between the years 2000 and 2005, after receiving approval from the Institutional Review Board of the respective hospitals. 23 patients (11 males) with genetically proven achondroplasia (Bellus et al. 1995, Niu et al. 1996) who were treated with lengthening were included in the study (group L). Mean age was 8.2 (5–13) years and mean height was 96 (87–103) cm. Only those patients who were followed to skeletal maturity were included, and patients who had complications after surgery such as intramedullary infection, bending, or refracture were excluded. The indication for surgery was any child of genetically proven achondroplasia who wanted surgery and who was fit for it. Since most of the patients were under 12 years of age, informed parental consent was taken after thorough counseling.

The control group (group C) was selected among children who were similar to the operated group in all respects except for the fact that they had not chosen surgery for financial, social, or other reasons. Group C comprised 12 children (6 males) with a mean age of 8.5 (5.4–14.5) years at the start of follow-up and with a mean height of 97 (88–103) cm. There was no statistically significant difference between the groups regarding age, sex, and height. The mean duration of follow-up was 9.8 (8.1–11.2) years.

### Operative and postoperative protocol

All patients in group L were operated on by the senior author, at two institutes between 2000 and 2005. They underwent bilateral tibial lengthening with monofocal proximal tibial osteotomy and the use of Ilizarov rings (Song et al. 2011). Distraction was started 7 days postoperatively at a rate of 0.25 mm 4 times a day until the desired length was achieved. The external fixator was removed when 3 new cortices were visible on plain radiographs (Fischgrund et al. 1994, Song et al. 2011).

### Evaluation of lengthening

Serial radiograms of the tibia were taken annually from the initial visit until the time of complete physeal closure, which was termed skeletal maturity and defined radiographically as the midpoint of a period during which the length did not change in 2 successive radiographic measurements (Chung et al. 2005). All lengths were measured in mm in a standardized fashion and calibrated using a 10-cm sized template for elimination of magnification errors.

### Parameters defined on the radiographs (Figures 1 and 2)

**Figure 1.**
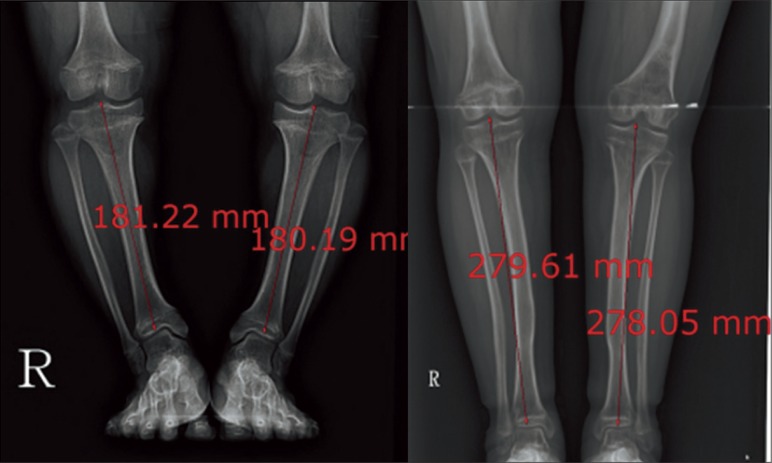
Measurement of tibial length in 2 patients of different ages. The length of the tibia was measured parallel to the long axis, from the most proximal part of the tibial eminence to the midpoint of the lowermost part of the tibial plafond.

**Figure 2.**
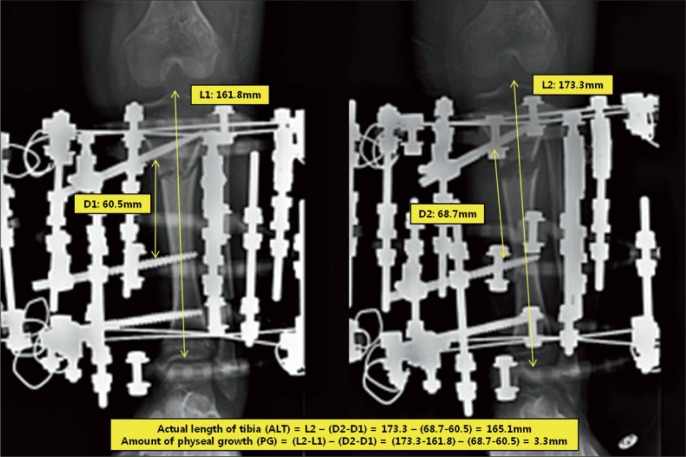
Radiographic measurement of the actual tibial length. Actual length of the tibia is calculated as L2 – (D2 – D1) and the amount of physeal growth is calculated as (L2 – L1) – (D2 – D1) where D1 is the distance between the middle half pins before distraction, D2 is the distance between the middle half pins after distraction, L1 is the length of the tibia before distraction, and L2 is the length of the tibia after distraction.

Total length of the tibia (TLT) was defined as the length of the tibia, and was measured on standing lower-extremity anterior-posterior radiographs parallel to the long axis from the uppermost portion of the tibial eminence to the midpoint of the lowermost portion of the tibial plafond (L1 and L2).

Actual length of the tibia (without distraction) (ALT) was defined as the amount of tibial growth attributed to the physis and measured by subtracting the amount of distraction (D2-D1) from the total length of the tibia (L2): ALT = (L2 – (D2 – D1)).

Amount of physeal growth (PG) was measured by subtracting the amount of distraction from the total increase in the length of the tibia (Chung et al. 2005): PG = ((L2 – L1) – (D2 – D1)).

Growth rate (GR) was determined from the ratio of the difference of the actual length of the tibia (ALT) of 2 successive radiographs divided by the time span in years (McCarthy et al. 2003): GR = (ALT_current_ – ALT_previous)_ / time span in years.

Percentage growth was determined by the ratio of the growth rate of group L divided by growth rate of group C: percentage growth = growth rate_group L_ / growth rate_group C_.

### Statistics

Data were clustered at each patient with repeated measures of total length of tibia, actual length of tibia, and the growth rate at each age between the 2 different groups. Thus, statistical significance was determined between groups for the total length of tibia, actual length of tibia, the change in growth rate, percentage growth, and laterality of the limb (right, left, of both) using linear mixed model. When employing the linear mixed model, we considered the covariance structure as compound symmetry of auto-regressive models of order 1. All of the statistical analyses, based on 2-sided test, were done using SAS software version 9.2. We regarded any p-value of < 0.05 to be statistically significant.

3 observers (2 pediatric orthopedic fellows and 1 orthopedic chief resident) measured each radiograph to test interobserver reliability for concurrence. Furthermore, each observer reviewed the radiographs twice within a 3-week period to test intraobserver reliability for reproducibility. Interobserver and intraobserver variability were assessed using the Spearman rank correlation test and an intraclass correlation coefficient. The data were analyzed using SPSS software version 16.0. Any p-value of < 0.05 was considered significant for the Spearman rank correlation. Intraclass correlation coefficients of 1 imply perfect agreement, and values of < 1 imply less than perfect agreement.

## Results

The intraobserver and interobserver reliability studies showed excellent reliability of measurements (with a correlation coefficient of between 0.92 and 0.96 and a p-value = 0.01) in our study. There was a high correlation between right and left side in each patient (p = 0.9), so we used the mean length of each side for each patient.

The mean total tibial lengths in the 2 groups were 163 mm and 165 mm on the primary radiographs (p = 0.7). In group L, the mean gain in tibial length was 9.2 (6.7–11.5) cm and the mean percentage gain in tibial length was 60% (41–70). The mean external fixator index was 1.3 (0.7–2.0) months/cm and the mean period of use of an external fixator was 9.8 (7.2–12.6) months. The mean total tibial lengths (TLT) at the time of skeletal maturity were 330 mm in group L and 277 mm in group C (p = 0.03). There was no statistically significant difference between males and females (p = 0.7).

**Figure 3.**
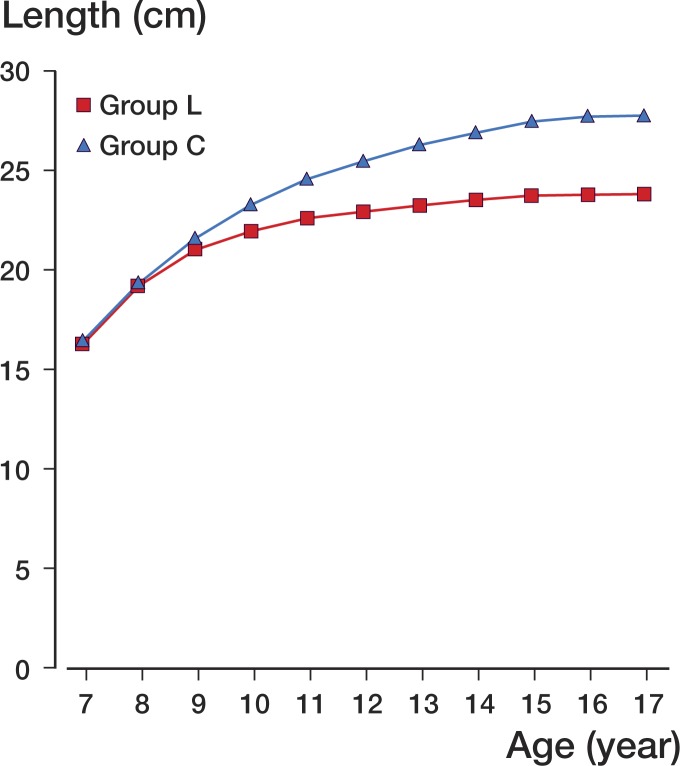
Mean actual tibial lengths in all groups.

In group L, skeletal maturity for the time of complete physeal closure was reached at 15.2 (SD 0.67) years while in group C it was reached at 16.0 (SD 0.54) years (p = 0.001). The mean ALT in group C showed a sustained gradual increase until skeletal maturity (without the pubertal spurt) with the final mean ALT being 277 mm. On the other hand, the ALT in group L showed a plateau especially after 11 years of age, the final mean ALT being 238 mm (p = 0.03) (Figure 3).

**Figure 4.**
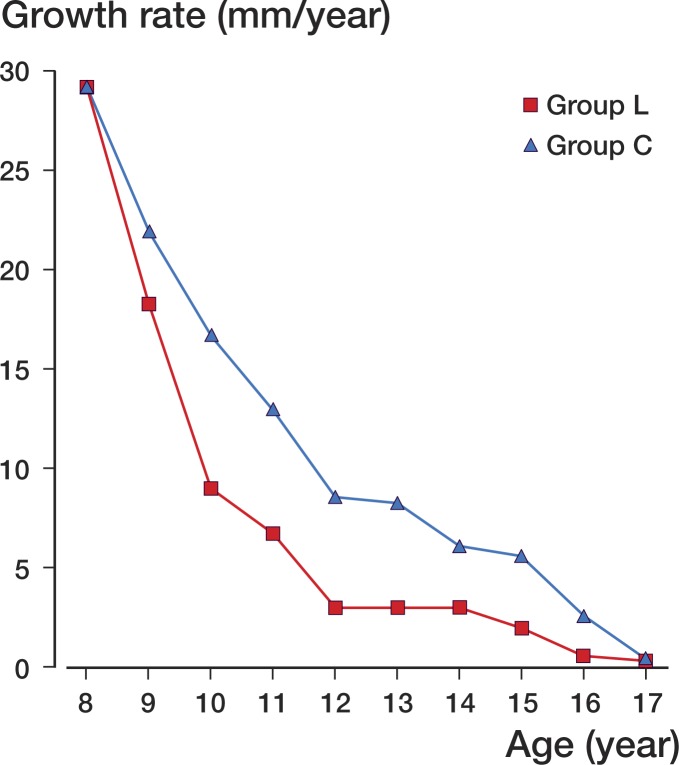
Curves with growth rate for both groups. Growth rate was determined from the ratio of the difference of the actual length of the tibia in two successive radiographs divided by the time span in years.

**Figure 5.**
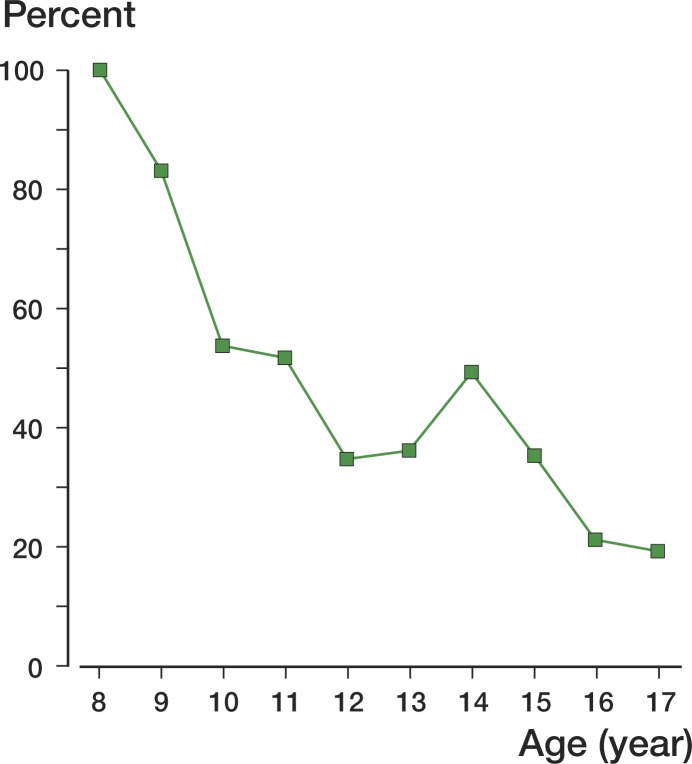
Percentage growth in lengthening group L as compared to that in control group C. Percentage growth was determined from the ratio of the growth rate in group L divided by growth rate in group C.

The patients in group C showed a maximum growth rate at the start of follow-up at 8 years of age, which progressively decreased over the years and became less than 10 mm/year after 11 years of age, until skeletal maturity was reached at 16 years of age (Figure 4). There was no significant difference in the growth rate between groups at the time of initial follow-up and until 2 years after surgery (p = 0.5), but after that it fell drastically. 2 years after surgery, around 11 years of age, the difference in growth rates in the groups became statistically significant and the percentage growth was 54% relative to group C (Figure 5).

In patients in group L, subgroup analysis according to amount of lengthening showed no statistically significant differences between each subgroup (Table).

**Table:** Result of subgroup analysis in lengthening group L

Group L subgroup	Amount of lengthening (%)	No. of patients	Age at skeletal maturity (SD)	Final actual tibial length (range)	Growth disturbance
A	∼40–50	2	15.1 (0.25) years	240 (216–273) mm	37.7 mm (14%)
B	∼50–60	18	15.2 (0.62) years	238 (213–268) mm	38.9 mm (14%)
C	∼60–70	3	15.4 (0.05) years	239 (238–240) mm	39.8 mm (14%)

There was no significant difference between each subgroup (p = 0.8).

**Figure 6.**
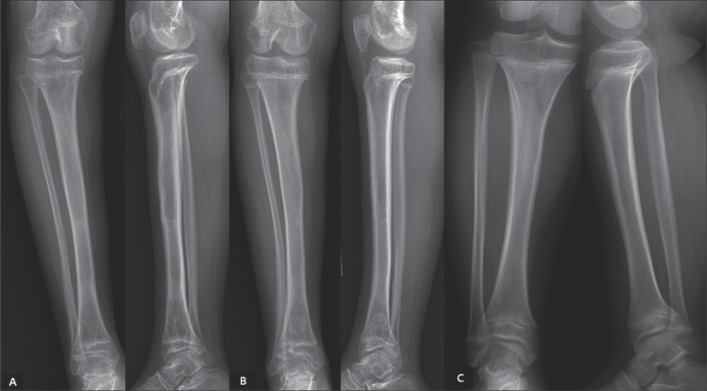
Characteristic patterns of physeal closure in 2 patients in group L (panels A and B) as compared to that for a patient of similar age in group C (panel C). The radiographs of a 13-year-old boy after 8.9 cm of lengthening (55%; panel A) and of a 12.8-year-old girl after 9.3 cm of lengthening (60%; panel B) showed premature closure of the lateral portion of the anterior proximal physis with relatively preserved distal physis. No physeal closure could be seen in the radiographs of a 13.1-year-old boy in group C (panel C).

**Figure 7.**
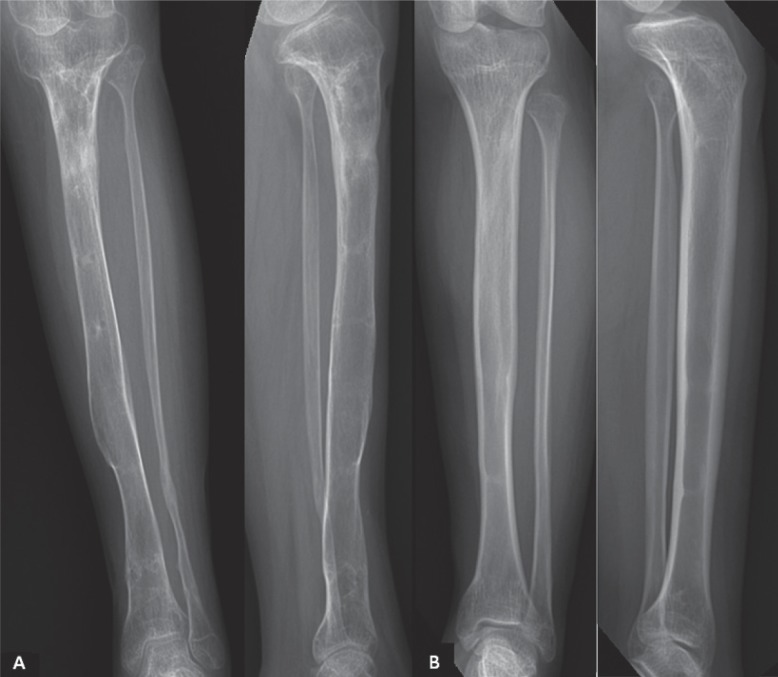
A 15.6-year-old girl after a 9.4 cm of lengthening (60%; panels labeled A) and a 16.2-year-old boy after 9.7 cm of lengthening (61%; panels labeled B) with complete closure of the proximal and distal physes.

A characteristic pattern of physeal closure was seen in the lengthened patients (Figure 6). Premature closure of the lateral part of the anterior tibial physis occurred in all patients; by 13 years of age, this part of the physis had fused in all group L patients. The medial portion of the proximal physis closed next. This part of the proximal tibial physis was closed prematurely in all patients, the average time of proximal medial physeal closure being 13.8 years. Generally, the distal tibial physis was not involved, with closure occurring at 16 years of age (Figure 7). No such pattern of physeal closure was seen in group C.

## Discussion

Bilateral limb lengthening has become common in achondroplasia (Aldegheri et al. 1988, Lavini et al. 1990, Vilarrubias et al. 1990, Saleh and Burton 1991, Ganel and Horoszowski 1996, Aldegheri and Dall'Oca 2001). It is not just a cosmetic adjustment of body height, but a tool to achieve normal body proportions in order to help these patients lead as normal a life as possible (Lavini et al. 1990).

Shapiro (1987) was one of the first to investigate longitudinal limb growth after lengthening. He described a series of 18 patients with lengthened femur and/or tibias of diverse etiology who were treated with the Wagner and Anderson techniques. The patients were followed up until skeletal maturity. He found a stimulation of femoral growth after femoral lengthening in 7 cases of congenitally short femurs, while the tibias showed significant retardation of growth. The growth retardation was 64% of normal, which is similar to the value of 54% that we found. Shapiro did not describe the chronology of growth inhibition, however—i.e. the period after lengthening when the growth inhibition was seen.

Sharma et al. (1996) noted growth retardation in their study of tibial lengthening in 7 cases of fibular hemimelia, causing total growth arrest in 2 patients. Sabharwal et al. (2000) confirmed the findings of Shapiro. On the other hand, McCarthy et al. (2003) did not find any effect of growth retardation in 19 patients after femoral and tibial lengthening. However, the amount of lengthening and lengthening percentage in that series was much less (6–7 cm and 15–25%) than in our series, which would probably explain these results.

The cause of growth inhibition and physeal damage after limb lengthening has been analyzed in animal models (Pennecot et al. 1983, Alberty et al. 1990, Albanese et al. 1996). These studies demonstrated that continuous compressive forces on the physis result in either temporary or permanent cessation of growth (the Heuter-Volkmann principle). They also showed that growth inhibition due to compressive forces applied across the growth plate is not an “all-or-none” or a “single-event” phenomenon, but rather a more gradual and sometimes reversible process that may recover after the compressive event is removed. This process may not be reversible under two conditions, however: when the physis itself is abnormal or dysplastic or when an abnormally large amount of compressive force is applied across the physeal plate. The first may be the reason for there not being much growth retardation after lengthening in fibular hemimelia where the distal tibial epiphysis is dysplastic, and the second may explain the findings in our series where a mean of 60% of lengthening was performed.

Another explanation was put forward by Trueta and Trias (1961), who showed that there is persistent damage to the blood supply to the parts of the physis that are most compressed, and this growth disturbance is directly proportional to the amount of compression applied. This may explain the characteristic involvement of the lateral portion of the proximal tibial physis in our series. Most of our patients with achondroplasia had genu varum deformity at the start of the treatment, which was corrected gradually through the fixator itself. This gradual correction would put an excessive amount of compressive force on the lateral side of the physis with relative distraction at the medial side, thus relatively speaking sparing the medial physis and affecting the lateral part of the physis.

Our series is the first to show the effects of lengthening on the growing physis in achondroplasia. The results of this group of patients are probably midway between the two extremes of hemimelia on the one hand, which shows a severe and sometimes sudden stoppage of physeal growth, and relatively benign conditions such as congenital hemiatrophy (with short femur and tibia) and poliomyelitis on the other hand, where physeal growth inhibition has been shown to be minimal and reversible (Shapiro 1987, Sharma et al. 1996, Sabharwal et al. 2000, McCarthy et al. 2003). Our series also gives a growth curve of the tibia in “normal” patients with achondroplasia in the Korean population. It can be seen in the curve that the growth rate in these patients dips to below 10 mm/year after 11 years of age. Thus, in order to have the least effect of physeal growth inhibition, we suggest that tibial lengthening in achondroplasia should be started after that age.

Our study had some limitations. Firstly, this was a small retrospective series with just a single diagnosis. Also, the parameters that were measured were purely radiographic without any clinical correlation. Secondly, we considered only tibial lengthening even though femoral lengthening is also a commonly performed procedure in achondroplasia. The reason for only studying tibial lengthening was that earlier studies had shown that the femur often escapes physeal insult. Also, in achondroplasia the tibia is the most deformed and is subject to the most changes during the lengthening process. Thirdly, we used only plain radiographs. Quantitative MR imaging is needed to study the physeal injury in detail (Ecklund and Jaramillo 2002).

In summary, we found that physeal damage occurs after limb lengthening by over 50% in achondroplasia. This damage is a gradual process that manifests itself about 2 years after surgery, and it is most pronounced in the anterior-lateral portion of the proximal tibial physis with sparing of the distal physis. In Korean children, lengthening should preferably be started at around 11 years of age.
